# Dual Immunohistochemistry Enhances Detection of Perineural Invasion in Oral Squamous Cell Carcinoma

**DOI:** 10.1111/odi.70172

**Published:** 2025-12-26

**Authors:** Túlio Silva Rosa, Lidiane de Paula Ribeiro, Caroline Alfaia Silva, Nicole Lonni, Daniella Serafin Couto Vieira, Filipe Modolo, Elena Riet Correa Rivero

**Affiliations:** ^1^ Postgraduate Program in Dentistry Federal University of Santa Catarina Florianópolis Brazil; ^2^ Department of Pathology, Health Sciences Center Federal University of Santa Catarina Florianopolis Brazil

**Keywords:** cancer diagnosis, dual staining, immunohistochemistry, oral squamous cell carcinoma, perineural invasion, tumor microenvironment

Perineural invasion (PNI) is a hallmark of tumor aggressiveness and a well‐established predictor of poor prognosis in oral squamous cell carcinoma (OSCC). Accurate detection of PNI is critical for tumor staging, surgical decision‐making, and post‐treatment surveillance (Li et al. 2021; Quintana et al. 2022; Kurtz et al. 2005). However, PNI remains challenging to identify, and its underrecognition can compromise prognostic evaluation and therapeutic planning. Conventional staining techniques, as hematoxylin and eosin (H&E) and single‐marker immunohistochemistry (IHC), often fail to provide sufficient contrast to accurately visualize tumor cells infiltrating or encasing nerve fibers, particularly in inflamed or limited stromal background (Kurtz et al. 2005). In addition, sampling variability, pathologist experience, tissue processing artifacts, and the limited number of histological sections examined are all potential sources of diagnostic inaccuracy of PNI (Binmadi and Basile 2011; Yan et al. 2021). These issues are particularly relevant in cases of subtle PNI, where focal perineural involvement or infiltration of small‐caliber nerve fibers may exhibit minimal morphological alterations, increasing the risk of oversight during routine evaluation (Binmadi and Basile 2011). Consequently, diagnostic accuracy and prognostic reliability of PNI assessments in OSCC may be compromised by underreporting, often resulting from incidental omission during standard histopathological evaluation. These challenges underscore the need for improved diagnostic strategies that enhance visualization and detection of PNI.

To address the diagnostic limitations of conventional staining, we employed a dual immunohistochemical method (DM) designed to simultaneously visualize epithelial and neural components in OSCC. This approach was applied to 62 OSCC cases from paraffin‐embedded samples. Histological sections were incubated with anti‐pancytokeratin (AE1/AE3; Dako; ready‐to‐use), followed by EnVision FLEX/HRP secondary detection and 3,3′‐diaminobenzidine (DAB) as chromogen (Dako, Carpinteria, CA, USA). Subsequently, slides were incubated with anti‐S100 (Dako; ready‐to‐use), visualized using the MACH 4 Universal AP Polymer Kit (Biocare Medical, Concord, CA, USA) and Vulcan Fast Red chromogen (Biocare Medical, Concord, CA, USA). This dual staining provided a clear contrast between tumor cells (brown) and nerve fibers (red) (Figure 1). The study was approved by the Human Research Ethics Committee (No. 17674419.9.0000.0121). All cases were also evaluated using conventional H&E staining.

**FIGURE 1:**
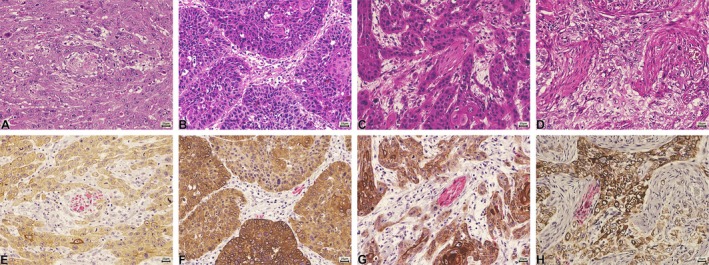
(A–D) Histological sections stained with hematoxylin and eosin showing perineural invasion. (E–H) Same sections shown above, but demonstrating perineural invasion by double staining with AE1/AE3 (malignant cells) and S100 (nerve).

Two examiners, blinded to all clinicopathological data, were calibrated for PNI assessment before analysis. Each examiner independently evaluated both H&E and DM slides, using an alternating sequence with 15 days between assessments to minimize recall bias. In each round, the presence or absence of PNI was recorded, and the diagnostic difficulty was rated using a Likert‐type scale.

The diagnostic performance of the two staining techniques was assessed in terms of PNI detection reliability, reproducibility, and repeatability. The DM outperformed H&E staining, showing significantly higher agreement for PNI identification (*κ* = 0.827–0.893 vs. 0.392–0.426), greater sensitivity (88.09%–92.85% vs. 50.0%–57.14%), perfect specificity (100% vs. 95.0%–100%), and superior overall accuracy (91.93%–95.16% vs. 66.13%–69.35%) (Table 1). Intraobserver agreement was also higher with the DM: observer A achieved *κ* = 0.803 (substantial to almost perfect), and observer B *κ* = 0.608 (moderate to substantial). In contrast, H&E yielded only moderate intraobserver agreement (*κ* = 0.556 and 0.638 for A and B, respectively). Interobserver agreement was superior with DM (*κ* = 0.796) compared to H&E (*κ* = 0.451). These findings highlight the improved diagnostic consistency and reliability of the DM, supported by enhanced sensitivity, specificity, and accuracy resulting from the simultaneous visualization of epithelial and neural structures.

**TABLE 1:** Comparison between histological methods: hematoxylin and eosin (H&E) versus the dual immunohistochemical method (AE1/AE3 and S100).

Evaluation method				
Classification	Hematoxylin and eosin Kappa value; 95% CI; [Agreement *n*, %]; *p*‐value		Dual method Kappa value; 95% CI; [Agreement *n*, %]; *p*‐value	
Intra‐observer variability^a^	(A1 versus A2)	(B1 versus B2)	(A1 versus A2)	(B1 versus B2)
	0.556; 0.344–0.768; [49, 79.03%]; *p* < 0.001	0.638; 0.434–0.842; [53, 85.48%]; *p* < 0.001	0.803; 0.656–0.950; [56, 90.32%]; *p* < 0.001	0.608; 0.410–0.806; [50, 80.65%]; *p* < 0.001
Inter‐observer variability^a^	A versus B		A versus B	
	0.451; 0.224–0.678; [48, 77.42%]; *p* < 0.001		0.796; 0.641–0.951; [56, 90.32%]; *p* < 0.001	

Comparison of perineural invasion detection		
PNI+	Hematoxylin and eosin Kappa value; 95% CI; [Agreement *n*, %]; *p*‐value	Dual method Kappa value; 95% CI; [Agreement *n*, %]; *p*‐value
A	0.426; 0.242–0.610; [43, 69.35%]; *p* < 0.001	0.893; 0.775–1.000; [59, 95.16%]; *p* < 0.001
B	0.392; 0.223–0.561; [41, 66.13%]; *p* < 0.001	0.827; 0.684–0.970; [57, 91.94%]; *p* < 0.001
Sensitivity	50.0%–57.14%	88.09%–92.85%
Specificity	95.0%–100%	100.0%
Accuracy	66.12%–69.35%	91.93%–95.16%
Difficulty level^b^	Difficult to moderate	Easy to moderate

*Note:* All assessments were performed independently.

Abbreviations: A, evaluator A; B, evaluator B; CI, confidence interval; PNI, perineural invasion.

^a^ Evaluations conducted with a 15‐day interval.

^**b**^ Likert scale.

Standard H&E staining is often insufficient and less sensitive for detecting PNI, particularly when it is subtle or focal, as in small‐caliber nerves or within densely desmoplastic stroma (Kurtz et al. 2005; Dhawan et al. 2016; Alves et al. 2022). In a previous study, H&E staining identified PNI in only 62% of OSCC cases, whereas the addition of S100 immunohistochemistry increased the detection rate to 82% (Kurtz et al. 2005). Consistent with this diagnostic enhancement, the *κ* values observed with the DM in our study demonstrated near‐perfect agreement between evaluators and the confirmed presence of PNI, exceeding the commonly accepted threshold of *κ* ≥ 0.80 for diagnostic reliability (McHugh 2012).

In a study on non‐melanoma skin cancers, Berlingeri‐Ramos et al. (2015) found that DM detected seven cases of cutaneous squamous cell carcinoma with PNI that were missed by H&E staining, all involving nerves ≤ 0.1 mm. Similarly, Donaldson and Weber (2019) demonstrated the value of dual staining with SOX10 and AE1/AE3 on frozen sections for real‐time PNI confirmation in diagnostically challenging diagnoses. These findings underscore the diagnostic advantage of simultaneous epithelial and neural labeling. Building on this evidence, our study validated the utility of dual AE1/AE3 and S100 immunolabeling in a single histological section, achieving over 91.93% accuracy and reinforcing its potential to enhance the precision and reliability of PNI detection.

Beyond enhancing the detection of PNI in malignant tumors, the DM may also aid in distinguishing histological findings that mimic true invasion, such as peritumoral fibrosis (Dunn et al. 2009). By improving the contrast between epithelial and neural components, this approach contributes not only to more accurate diagnosis but also to reduced false‐positive interpretation in challenging contexts. This distinction is clinically relevant, since benign conditions may closely resemble true PNI, potentially leading to misdiagnosis if not carefully assessed (Dunn et al. 2009).

From a practical perspective, the method is technically feasible and easily adaptable to routine pathology workflows, as it employs widely available antibodies and chromogens. However, the DM involves higher costs and longer processing times compared to single IHC or routine H&E (Appendix S1).

Accurate PNI significantly affects the locoregional recurrence and survival in patients with OSCC (Li et al. 2021). The use of objective histopathologic criteria, therefore, enhances carcinoma grading and supports more precise prognostic and therapeutic decisions. As a complementary tool for laboratories already equipped for immunohistochemistry, DM improves tumor–nerve visualization and enables faster, more reliable detection of PNI, particularly in cases where H&E results are inconclusive.

DM also offers several advantages in cancer research by enabling the simultaneous visualization of two distinct cellular or tissue components within the same histological section. This approach can be applied to investigate processes associated with PNI, such as the role of epithelial–mesenchymal transition in its underlying mechanisms.

## Author Contributions

**Túlio Silva Rosa:** conceptualization, investigation, methodology, formal analysis, writing – original draft, software. **Lidiane de Paula Ribeiro:** methodology, investigation, formal analysis, writing – original draft. **Caroline Alfaia Silva:** methodology, validation, investigation, writing – review and editing. **Nicole Lonni:** investigation, validation, writing – review and editing, software. **Daniella Serafin Couto Vieira:** writing – review and editing, conceptualization, resources, visualization. **Filipe Modolo:** conceptualization, writing – review and editing, validation. **Elena Riet Correa Rivero:** conceptualization, methodology, writing – review and editing, project administration, funding acquisition, visualization, supervision.

## Conflicts of Interest

The authors declare no conflicts of interest.

## Supporting information


**Appendix S1:** Comparisons between the techniques for the identification of perineural invasion (PNI).

## Data Availability

The data that supports the findings of this study is available in the Supporting Information of this article.
