# Supplementary material for: Dual Immunohistochemistry Enhances Detection of Perineural Invasion in Oral Squamous Cell Carcinoma
Source: Oral Dis. 2025 Dec 26;32(3):684–7. doi: 10.1111/odi.70172 (PMC13125729; doi:10.1111/odi.70172)
Supplement: Supplementary file 1 — Appendix S1: Comparisons between the techniques for the identification of perineural invasion (PNI). [file ODI-32-684-s001.pdf]

**Appendix S1. Comparisons between the techniques for the identification of Perineural invasion (PNI).**

| Technique | Workflow | Key advantages | Limitations |
|---|---|---|---|
| **H&E**<br>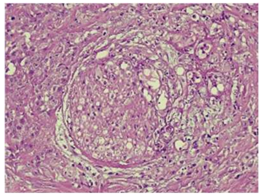 | **1.** Deparaffinization and Rehydration<br>**2.** Hematoxylin Staining<br>**3.** Eosin Staining<br>**4.** Dehydration and Clearing<br>**5.** Slide Mounting<br>**Processing Time:** 1h. | • Short processing time.<br>• Widely available in diagnostic laboratories.<br>• Good for assessing general tissue morphology.<br>• Low cost. | • Provides limited molecular information.<br>• Not detect specific proteins.<br>• Difficult to identify PNI in cases of focal perineural involvement, infiltration of small-caliber nerve fibers, or intense stromal inflammation. |
| **IHC (single-marker)**<br>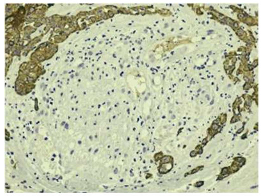<br>Pancytokeratin<br>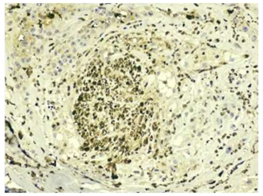<br>S100 | **1.** Deparaffinization and Rehydration<br>**2.** Antigen retrieval<br>**3.** Endogenous peroxidase blockade<br>**4.** Primary antibody incubation<br>**5.** Secondary antibody (EnVision FLEX/HRP)<br>**6.** Chromogen - DAB<br>**7.** Counterstaining - Harris hematoxylin<br>**8.** Dehydration and Clearing<br>**9.** Slide Mounting<br>**Processing Time:** 3-4h. | • Detects specific proteins.<br>• Provides spatial localization of the marker within the tissue. | • Requires specific antibodies.<br>• More expensive than H&E.<br>• Longer processing time compared to H&E.<br>• Cannot evaluate two markers simultaneously.<br>• Provides insufficient contrast to accurately visualize tumor cells infiltrating or encasing nerve fibers, particularly in cases of focal perineural involvement, infiltration of small-caliber nerve fibers, or intense stromal inflammation. |
| **Dual IHC (two-markers)**<br>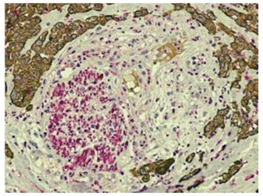<br>Pancytokeratin (DAB)<br>S100 (VFR) | **1.** Deparaffinization and Rehydration<br>**2.** Antigen retrieval<br>**3.** Endogenous peroxidase blockade<br>**4.** FIRST primary antibody incubation<br>**5.** Secondary antibody (EnVision FLEX/HRP)<br>**6.** Chromogen - DAB<br>**7.** SECOND primary antibody incubation<br>**8.** Secondary antibody (MACH4 Universal)<br>**9.** Polymer (MACH AP Polymer)<br>**10.** Chromogen - VFR<br>**11.** Counterstaining - Harris hematoxylin<br>**12.** Dehydration and Clearing<br>**13.** Slide Mounting<br>**Processing Time:** 5-6h. | • Allows simultaneous visualization of multiple markers.<br>• Improves tumor–nerve visualization<br>• Identification of PNI, is faster and more reliable even in cases with focal perineural involvement or infiltration of small-caliber nerve fibers, or with intense stromal inflammation. | • More complex technique.<br>• Requires optimization.<br>• More expensive than H&E and IHC (single-marker).<br>• Longer processing time compared to H&E and IHC. |

Legend: DAB, 3,3'-diaminobenzidina; H&E, Hematoxylin and Eosin Staining; IHC, Immunohistochemistry; PNI, Perineural invasion; VFR, Vulcan Fast Red.
